# New Real-Time Impulse Noise Removal Method Applied to Chest X-ray Images

**DOI:** 10.3390/diagnostics12112738

**Published:** 2022-11-09

**Authors:** Nasr Rashid, Kamel Berriri, Mohammed Albekairi, Khaled Kaaniche, Ahmed Ben Atitallah, Muhammad Attique Khan, Osama I. El-Hamrawy

**Affiliations:** 1 Department of Electrical Engineering, College of Engineering, Jouf University, Sakaka 72388, Saudi Arabia; 2 Department of Electrical Engineering, Faculty of Engineering, Al-Azhar University, Nasr City, Cairo 11884, Egypt; 3 LAMMDA Laboratory, University of Sousse, Sousse 4054, Tunisia; 4 LETI, ENIS, University of Sfax, Sfax 3038, Tunisia; 5 Department of CS, HITEC University, Taxila 47080, Pakistan; 6 Electrical Engineering Department, Faculty of Engineering, Suez Canal University, Ismailia 41522, Egypt

**Keywords:** MLVMF, impulsive noise, image denoising, chest X-ray images, cardiovascular diseases diagnosis, HLS, FPGA

## Abstract

In this paper, we propose a new Modified Laplacian Vector Median Filter (MLVMF) for real-time denoising complex images corrupted by “salt and pepper” impulsive noise. The method consists of two rounds with three steps each: the first round starts with the identification of pixels that may be contaminated by noise using a Modified Laplacian Filter. Then, corrupted pixels pass a neighborhood-based validation test. Finally, the Vector Median Filter is used to replace noisy pixels. The MLVMF uses a 5 × 5 window to observe the intensity variations around each pixel of the image with a rotation step of π/8 while the classic Laplacian filters often use rotation steps of π/2 or π/4. We see better identification of noise-corrupted pixels thanks to this rotation step refinement. Despite this advantage, a high percentage of the impulsive noise may cause two or more corrupted pixels (with the same intensity) to collide, preventing the identification of noise-corrupted pixels. A second round is then necessary using a second set of filters, still based on the Laplacian operator, but allowing focusing only on the collision phenomenon. To validate our method, MLVMF is firstly tested on standard images, with a noise percentage varying from 3% to 30%. Obtained performances in terms of processing time, as well as image restoration quality through the PSNR (Peak Signal to Noise Ratio) and the NCD (Normalized Color Difference) metrics, are compared to the performances of VMF (Vector Median Filter), VMRHF (Vector Median-Rational Hybrid Filter), and MSMF (Modified Switching Median Filter). A second test is performed on several noisy chest x-ray images used in cardiovascular disease diagnosis as well as COVID-19 diagnosis. The proposed method shows a very good quality of restoration on this type of image, particularly when the percentage of noise is high. The MLVMF provides a high PSNR value of 5.5% and a low NCD value of 18.2%. Finally, an optimized Field-Programmable Gate Array (FPGA) design is proposed to implement the proposed method for real-time processing. The proposed hardware implementation allows an execution time equal to 9 ms per 256 × 256 color image.

## 1. Introduction

The processes of creating, acquiring, saving, and transmitting images often generate noise. A random alteration that an image undergoes can change the intensity of certain pixels to the minimum or maximum value, either 0 or 255. This phenomenon, known as impulsive noise or “salt and pepper” noise, can appear in a digital image due to data transmission errors, the presence of fine particles on the sensor elements of the camera, or faulty memory locations in the storage hardware. The presence of such noise considerably affects the process of understanding the image. Edge detection and segmentation are thus biased. This is the reason why work dedicated to the filtering and denoising of images has, for several years, taken an important place in the field of computer vision.

Various filters for removing noise have emerged. Several approaches have been followed and validated. We can mainly cite the basic vector filters [1,2,3,4,5,6,7], the weighted vector filters [8,9], the adaptive vector filters [10,11,12,13,14,15,16,17], the hybrid vector filters [18,19], the fuzzy vector filters [20,21,22], the neural network based vector filters [23,24,25,26], and the morphological based vector median filters [27,28]. The choice of a method remains dependent on the application. In this work, we are looking for a real-time architecture capable of restoring the chest x-ray images as well as possible. This will allow experts to better diagnose possible pathologies such as cardiovascular diseases, asthma, lung cancer, pneumonia, COVID-19, and tumors [29]. Maximum restoration quality also improves the performance of recognition algorithms based on deep learning, multi-channel CNN architecture or angle transformation, which has become very popular in recent years [30,31,32,33,34,35,36].

Among the families of filters mentioned above, the basic vector filters, the adaptive vector filters, and the hybrid vector filter are best suited for real-time implementation [17,18,19,37,38,39,40]. These filters are not greedy in terms of resources. This generally involves applying one or more simple heuristics and performing a noisy image scan by calculating a measurement for each pixel (a normalized sum of multiplications), making it possible to distinguish the very strong frequency variations. Methods based on VMF (Vector Median Filter) have always shown very good behavior in the case of low percentage impulsive noise. Methods based on AVMF (Adaptive Vector Median Filter), as well as HVF (Hybrid Vector Filter), are more complex to implement but far better in the case of high percentage impulsive noise [41]. Cited families (based on VMF, AVMF, or HVF) look, firstly, for corrupted pixels: we talk about noisy pixels identification. This step is very important and determines the quality of the image restoration. If some noisy pixels are not identified, they remain unchanged in the final result and disrupt the comprehension process. Otherwise, if non-noisy pixels are identified as being noise, they will be replaced, which negatively affects the quality of the restored image.

In this paper, we propose a new approach to refine the search and identification of noisy pixels. Considering a 5 × 5 window, our proposed MLVMF uses a modified Laplacian filter to observe the intensity variations around each pixel of the image with a rotation step of π/8 while the classic Laplacian filters often use rotation steps of π/2 or π/4 [42,43,44,45]. Reducing the angle of rotation around the pixel makes it possible to take advantage of additional information about its neighborhood. MLVMF does not stop at this stage. Identification of corrupted pixels can still fail against a high percentage of impulsive noise. Indeed, two or more noisy pixels having the same intensity (0 or 255) can be 4-connected or 8-connected. The local second derivative around this kind of pixel will not be able to detect strong variations. Once the noisy pixels are detected thanks to the new proposed filters, then eliminated by a simple VMF, a second round is initiated to search for the adjacent noisy pixels. We propose a second set of filters calculating, in eight directions, the intensity variations around each pixel of the image without taking into account the eight direct neighbors.

To validate our method, MLVMF is tested on standard images with a noise percentage varying from 3% to 30%. Obtained performances in terms of PSNR (Peak Signal to Noise Ratio) and NCD (Normalized Color Difference) are compared to the performances of VMF (Vector Median Filter) [1], VMRHF (Vector Median-Rational Hybrid Filter) [40], and MSMF (Modified Switching Median Filter) [46]. The results obtained show the effectiveness of the proposed approach.

The third and last contribution in this paper is the hardware implementation of the proposed nonlinear approach. Research has adopted hardware acceleration as a solution to reduce implementation complexity. In [47], using two hardware architectures, authors implement standard and multi-level median filters. According to [48], a novel 3 × 3 window median filtering algorithm is developed based on a bit-serial sorting algorithm with a high speed of operation and low hardware complexity. In [40], a hardware implementation of the VMRHF for color images is described. The relational function implementation is simplified using some approximations in this hardware architecture. To implement VMF efficiently, authors in [49] suggest a fast parallel architecture. VMF filter implementations in this architecture use approximation to implement L2 norms. However, these hardware architectures need more development time and lack the adaptability of design upgrading. In fact, Low-Level Synthesis (LLS) using Hardware Description Language (HDL) on an FPGA circuit is used to build and implement these systems. With LLS design, the Register Transfer Level (RTL) description can be modified to produce an excellent, efficient netlist. However, creating such an RTL description takes a lot of work and time, especially for complicated applications [50,51,52]. This is because each low-level circuit’s activities must be described. Nevertheless, only hardware designers with specialized knowledge and abilities are capable of creating a sophisticated system. Therefore, in order to decrease the complexity of the FPGA design, it is imperative to go from Low-Level Synthesis (LLS) to High-Level Synthesis (HLS) [53,54,55]. The HLS tool synthesizes the formalized algorithms into a behavioral and structural RTL hardware description utilizing software high-level languages (systemC, C/C++, etc.). Thereby, several academic and commercial HLS tools are developed, such as Xilinx Vivado HLS, Intel OpenCL, Catapult-C, and ROCCC. Thus, our objective in this part is to use HLS flow to generate various hardware architectures for our proposed denoising algorithm. These architectures are designed in terms of FPGA cost and execution time. The best-designed architecture will be implemented and validated on the Xilinx Zynq FPGA.

This paper is organized as follows. In Section 2, we present the proposed denoising algorithm (MLVMF) by detailing its different stages as well as the filters used. To validate the proposed method, Section 3 shows the MLVMF results tested on standard images with a noise percentage varying from 3% to 30%. Obtained performances in terms of processing time, as well as image restoration quality through the PSNR and the NCD measurement, are compared to the performances of VMF, VMRHF, and MSMF. An additional test is performed on noisy chest x-ray images used in cardiovascular disease diagnosis as well as COVID-19 diagnosis. Finally, the HLS designs for the MLVMF are described in Section 4.

## 2. Modified Laplacian Vector Median Filter—MLVMF

A noisy pixel is always associated with a high frequency in its direct neighborship. Impulsive noise is no exception to this rule. To identify a noisy pixel, it is, therefore, necessary to calculate the variations in intensity locally. A double derivative around the pixel makes it possible to measure this variation: this is the Laplacian operator. Let I be an image of size n × m. Let (*x*,*y*) be the position of a pixel in the image *I*. The Laplacian L(x,y) of an image with pixel intensity values I(x,y) is given by:

(1) $$L\left(x,y\right)=\frac{\partial^{2}I}{\partial x^{2}}+\frac{\partial^{2}I}{\partial y^{2}}\;$$

**Figure 1:**
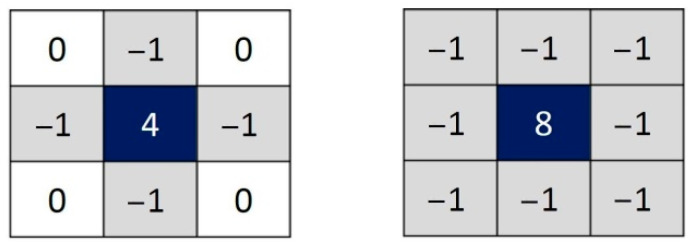
Two commonly used discrete approximations to the Laplacian filter.

Since the input image is represented as a set of discrete pixels, we have to find a discrete convolution kernel that can approximate the second derivatives in the definition of the Laplacian. Two commonly used small kernels are shown in Figure 1.

**Figure 2:**
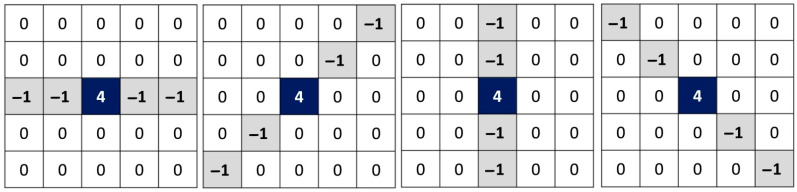
Four commonly used kernels in the case of impulsive noise identification. Angle of rotation (from left to right): 0, π/4, π/2, 3π/4.

When searching for and identifying noisy pixels of the impulsive type, there is a tendency to widen the Laplacian filters. In general, these filters are of size 5 × 5 instead of 3 × 3. This is dictated by the concern to ensure that it is indeed an isolated pixel in terms of intensity. For these same reasons and contrary to the search for edges, the convolution is not conducted all at once but rather in several directions around the pixel. Figure 2 shows the four commonly used kernels in the case of impulsive noise identification.

**Figure 3:**
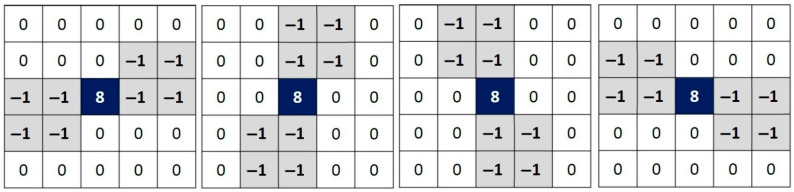
Proposed four new kernels according to the directions π/8, 3π/8, 5π/8, and 7π/8.

As shown in Figure 2, the rotation step of the Laplacian is equal to $\frac{\pi }{4}$: kernel $K_{1}$ at 0, kernel $K_{2}$ at $\frac{\pi }{4}$, kernel $K_{3}$ at $\frac{\pi }{2}$, and kernel $K_{4}$ at $\frac{3\pi }{4}$. A smaller rotation step allows a deeper investigation of the neighborhood of a pixel. However, the rectangular aspect of the pixels and the small size of the search window show no more than four discernible directions. Increasing the size of the window will increase the calculation time. It is nevertheless possible to approximate new directions. We propose, in Figure 3, four new kernels in order to estimate the variations of intensity according to the directions $\frac{\pi }{8}$, $\frac{3\pi }{8}$, $\frac{5\pi }{8}$, and $\frac{7\pi }{8}$. New kernels are: kernel $K_{5}$ at $\frac{\pi }{8}$, kernel $K_{6}$ at $\frac{3\pi }{8}$, kernel $K_{7}$ at $\frac{5\pi }{8}$, and kernel $K_{8}$ at $\frac{7\pi }{8}$.

**Figure 4:**
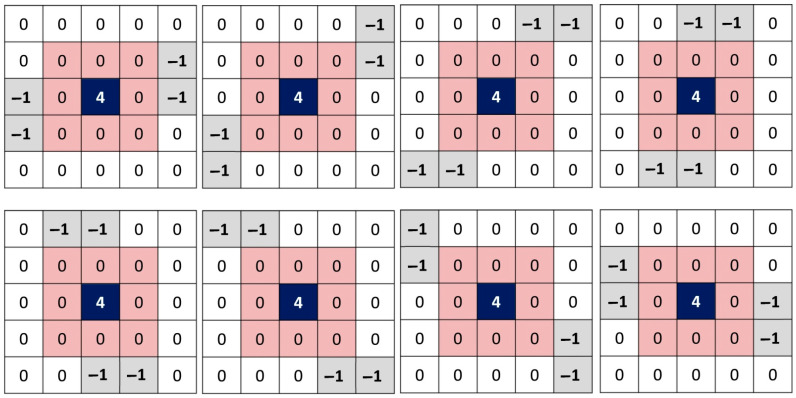
Eight new kernels proposed without taking into account the eight direct neighbors, $K_{9}$ to $K_{16}$.

Identification of corrupted pixels can still fail against a high percentage of impulsive noise. Indeed, two or more noisy pixels having the same intensity (0 or 255) can be 4-connected or 8-connected. The local second derivative around this kind of pixel will not be able to detect strong variations. We propose a second set of filters calculating in eight directions the intensity variations around each pixel of the image without taking into account the eight direct neighbors. Figure 4 shows the proposed eight kernels: $K_{9}$ to $K_{16}$.

The calculation of the variation is carried out as follows. For each position (x, y) in the image I, we compute the absolute value of the convolution product noted $V_{ij}$ between the kernel $K_{i}$ and the image $I_{j}$.

(2) $$V_{ij}\left(x,y\right)=\left|K_{i}⊗\;I_{j}\right|,$$

where j varies from 1  to 3, such as $I_{1}$ is the image red component, $I_{2}$ is image green component, and $I_{3}$ is the image blue component. A first scan of the image is performed with filters $K_{1}$ to $K_{8}$. For each image pixel, we, therefore, obtain 24 measurements of variation. To judge the importance of the intensity variation around a pixel (x,y), we observe the minimum value $M_{1}\left(x,y\right)$ among the 24 measurements obtained.

(3) $$M_{1}\left(x,y\right)=\min[V_{ij}\left(x,y\right),\;i=1..8,\;j=1..3],$$

If $M_{1}\left(x,y\right)$ exceeds a predefined threshold T then the pixel (x,y) is considered a candidate to be an impulsive noise. At this precise moment, we retrieve the color layer of the image giving this minimum measurement and declare the pixel as noise if its intensity at this layer is 0 or 255.

$$M_{1}\left(x,y\right)=\min[V_{ij}\left(x,y\right),\;i=1..8,\;j=1..3]\;if\;M_{1}\left(x,y\right)>T\;then\;\left(i_{ind},j_{ind}\right)=index\left(\min[V_{ij}\left(x,y\right),\;i=1..8,\;j=1..3\right])\;if\;I_{j_{ind}}\left(x,y\right)=0\;or\;255\;then\;pixel\;\left(x,y\right)is\;an\;impulsive\;noise$$

To eliminate the noisy pixel, we use the VMF method [1]. The image obtained and noted $I_{c}$ is then scanned again. This scan is performed with filters $K_{9}$ to $K_{16}$.

(4) $$V_{ij}\left(x,y\right)=\left|K_{i}⊗I_{cj}\right|,\;\;\;\;\;\;i=9..16,$$

where j varies from 1  to 3, such as $I_{c1}$ is the image red component of $I_{c}$, $I_{c2}$ is image green component of $I_{c}$, and $I_{c3}$ is the image blue component of $I_{c}.$

For each image pixel, we also obtain 24 measurements of variation. We observe the minimum value $M_{2}\left(x,y\right)$ among the 24 measurements obtained.

(5) $$M_{2}\left(x,y\right)=\min[V_{ij}\left(x,y\right),\;i=9..16,\;j=1..3],$$

**Figure 5:**
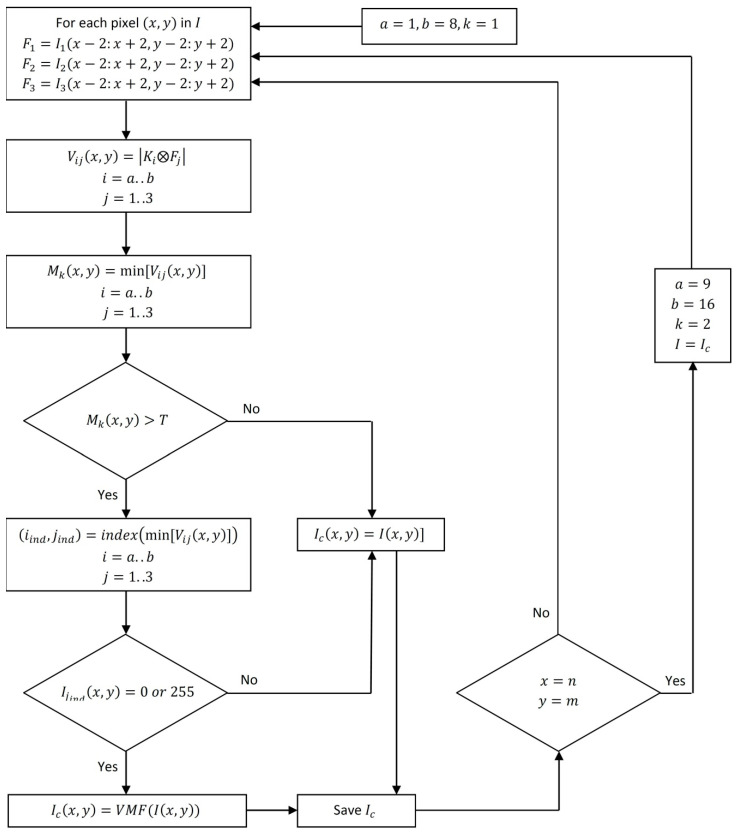
MLVMF flowchart.

To declare a pixel as noise, we proceed in the same way as for the first scan using the same value of predefined threshold  T. To eliminate the noisy pixel, we use the VMF method [1]. Figure 5 shows the MLVMF flowchart.

## 3. MLVMF Performances

The proposed MLVMF filter is assessed in terms of image restoration quality and execution time to remove the impulsive “salt and pepper” in color images. We compare MLVMF performances to the performances of VMF, VMRHF, and MSMF filters. The metrics used in this comparative study are: The Peak Signal to Noise Ratio (PSNR) and the Normalized Color Difference (NCD). PSNR is defined by (6)–(8).

(6) $$PSNR=10\;\log\left(\frac{255^{2}}{MSE}\right),$$

(7) $$MSE_{l}=\frac{1}{nm}\sum_{i=1}^{n}\sum_{j=1}^{m}{\left(I_{l}\left(i,j\right)-I_{cl}\left(i,j\right)\right)}^{2},$$

(8) $$MSE=\frac{MSE_{r}+MSE_{g}+MSE_{b}}{3},$$

where l the index of the color channel of a color image (r=red, g=green, b=blue). I represents the original image. $I_{c}$ is the filtered image. n is the number of rows of an image and m the number of columns.

Color distortion is estimated using the NCD, which is defined by (9).

(9) $$NCD=\frac{\sum_{i=1}^{n}\sum_{j=1}^{m}\sqrt{{\left(Y\left(i,j\right)-Y_{c}\left(i,j\right)\right)}^{2}+{\left(U\left(i,j\right)-U_{c}\left(i,j\right)\right)}^{2}+{\left(V\left(i,j\right)-V_{c}\left(i,j\right)\right)}^{2}}}{\sum_{i=1}^{n}\sum_{j=1}^{m}\sqrt{{\left(Y\left(i,j\right)\right)}^{2}+{\left(U\left(i,j\right)\right)}^{2}+{\left(V\left(i,j\right)\right)}^{2}}},$$

where Y represents the original image luminance, $Y_{c}$ is the filtered image luminance, U and V are original image chrominance components, $U_{c}$ and $V_{c}$ are filtered image chrominance components.

**Figure 6:**
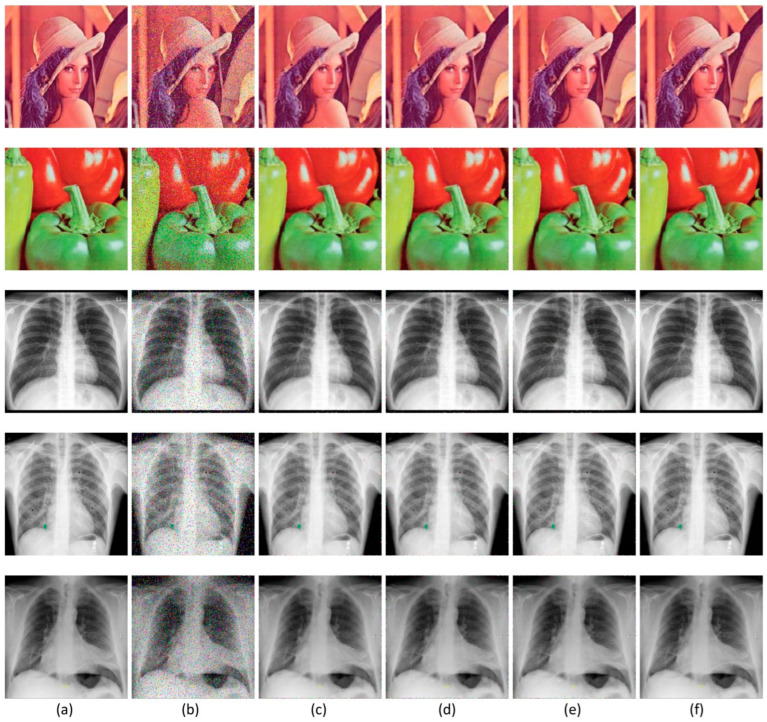
(**a**) Original images. (**b**) Corrupted images with 10% impulsive noise. Filtered images using (**c**) VMF, (**d**) VMRHF, (**e**) MSMF, and (**f**) MLVMF filters.

The proposed MLVMF is developed using C/C++ programming language, compiled by Visual C++ 2010 software tool. Execution is performed under Core™i7-1165g7@2.80ghz Intel processor. The processor timer is used to determine the execution time. We choose five test images (Figure 6), including two standard images (Lena and Peppers) and three chest x-ray images (CXR1, CXR2, and CXR3) [56]. All images used are 256×256 in size. Test images are corrupted by various levels of “salt and pepper” impulsive noise. These levels are 3%, 5%, 10%, 20%, and 30%. Based on several experimental tests, we set the threshold T at 75.

**Figure 7:**
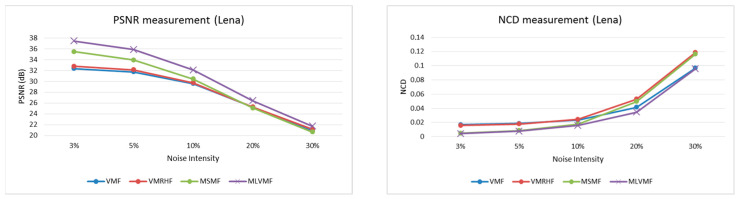

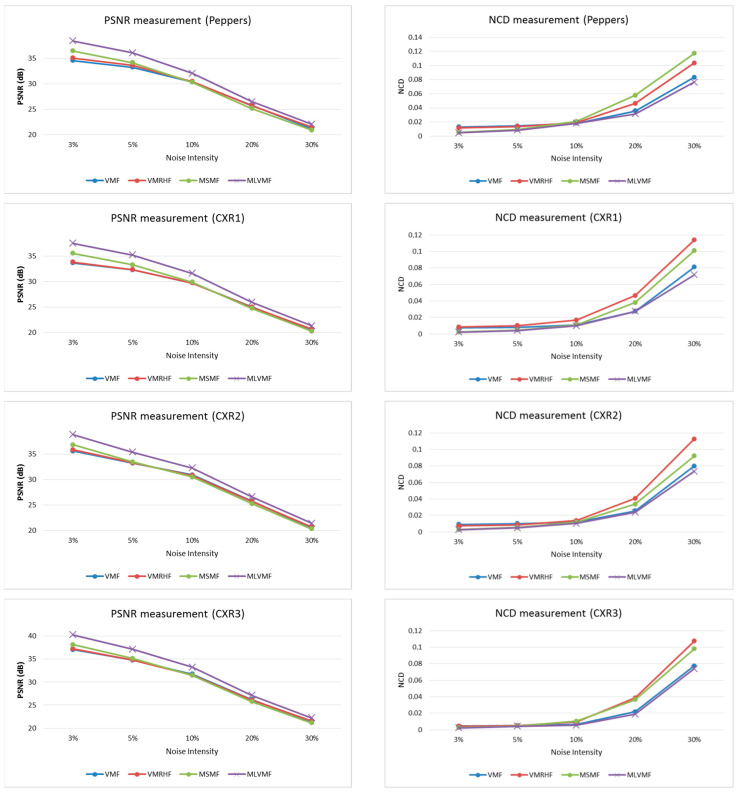
Performance comparison of the VMF, VMRHF, MSMF, and MLVMF filters using Lena, Peppers, CXR1, CXR2, and CXR3 images for various levels of impulsive noise. Left column: PSNR(dB) performance comparison. Right column: NCD performance comparison. Impulsive noise levels are: 3%, 5%, 10%, 20%, and 30%.

Table 1 shows the values of quality measurements according to PSNR and NCD after the filtering process performed by VMF, VMRHF, MSMF, and proposed MLVMF filters. According to this table, we can notice that the MLVMF is more efficient than the other three filters. Compared to the MSMF, which has the best performance among the three filters in question in terms of PSNR, the MLVMF provides a high PSNR value of 5.5%. Compared to the VMF, which has the best performance among the same three filters in terms of NCD, the MLVMF provides a low NCD value of 18.2%. Moreover, subjective measurement confirms the efficiency of our proposed MLVMF. Figure 6 confirms that the filtered images (Lena, Peppers, CRX1, CRX2, and CRX3) obtained after MLVMF processing are too close to the original images, thanks to a better quality of restoration. Figure 7 shows the comparison of performances measured in terms of PSNR and NCD metrics using Lena, Peppers, CRX1, CRX2, and CRX3 images for various levels of impulsive noise (3%, 5%, 10%, 20%, and 30%). In addition to the fact that the MLVMF is clearly superior in the case of a low and medium noise percentage, the proposed filter is clearly distinguished from other filters when the noise is high. This mainly comes down to the investigation carried out by the filters K9 to K16 and which succeeded in identifying the adjacent corrupted pixels. The execution time of the proposed MLVMF under Core™i7-1165g7@2.80ghz Intel processor reaches 1483 ms per 256×256 color image. To decrease this execution time and allow real-time calculation, Section 4 describes a proposed High-Level Synthesis (HLS) design for the MLVMF filter.

## 4. High-Level Synthesis (HLS) Designs for the MLVMF Filter

The HLS flow used with FPGA is an important tool for engineers to quickly explore the design space from a given behavioral description based on a high-level programming language (e.g., SystemC, C/C++). Because of this, the HLS has become a useful and effective tool for boosting design productivity and decreasing design cycle time. Various HLS tools have been developed in this regard, such as the Xilinx Vivado HLS tool, which offers a number of directives to produce an optimum hardware design for any algorithm. In fact, the RESOURCE directive allows the arrays to be implemented as registers or memories. Additionally, the ALLOCATION directive can be used to optimize how the arithmetic operation is implemented. Furthermore, using the PIPELINE and UNROLL directives, the loops can be pipelined, not unrolled, or fully/partially unrolled to increase the loop iterations’ performance (i.e., to reach a higher throughput).

**Figure 8:**
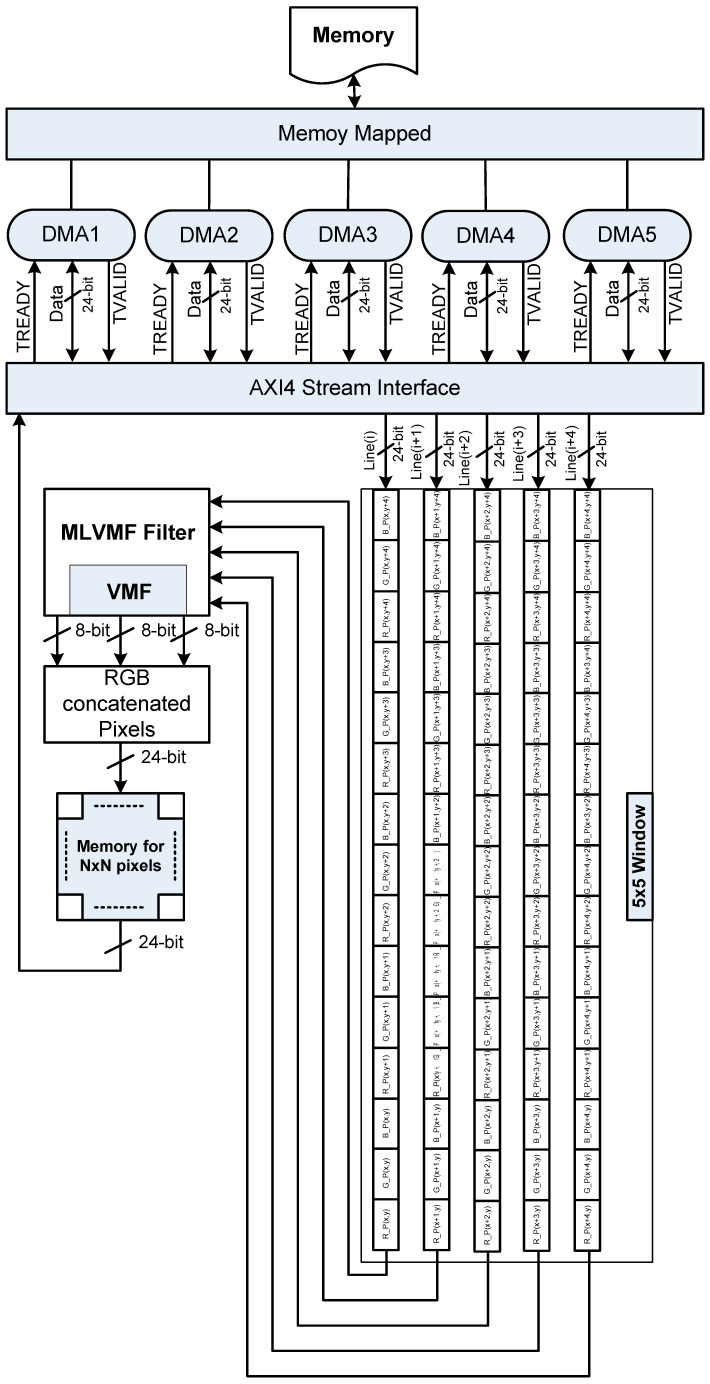
Proposed MLVMF filter block diagram.

Figure 8 shows the block diagram of the hardware architecture generated for the MLVMF filter. The generation process is performed by Xilinx Vivado HLS 18.1 tool from a specific C/C++ code. Our architecture uses 5 DMA (Direct Memory Access) to transfer five image lines in parallel in order to increase the throughput of our MLVMF filter. The hardware architecture of the proposed MLVMF is based on a 5 × 5 modified Laplacian filter and a 3 × 3 VMF filter. Each denoising RGB pixel (in 24 bit) is concatenated and stored in 256 × 256 × 24-bit internal memory. To optimize the architecture, some directives (such as PARTITION and PIPELINE) are incrementally introduced to the MLVMF C/C++ code. So, we produce various MLVMF hardware designs. We then retain the optimized design, which gives a compromise between hardware cost and processing time. The hardware requirements in terms of Lookup-Table (LUT), Flip-Flops (FF), BRAM blocks, and DSP blocks for the various MLVMF designs on the Zynq XCZU9EG FPGA are shown in Table 2.

In Table 2, Solution 1 presents the hardware architecture without any optimization. It is clear that this solution is not greedy in terms of resources but requires a very large number of clock cycles to filter the entire image. With a clock frequency equal to 115 MHz, Solution 1 needs 1810 ms per 256 × 256 color image. In Solution 2, we propose to apply the PIPELINE directive. Hardware cost increases but is still well below the maximum capacities of the hardware. On the other hand, the number of clock cycles decreases by 97.5%. An image can, therefore, be processed in 46 ms. With the aim of further reducing the number of clock cycles, we add in Solution 3 the PARTITION directive, which allows splitting the filtering window (Figure 8) into small blocks and promoting parallel access to data. Thank to Solution 3, we reach a computation time equal to 9 ms, i.e., 200 times faster than Solution 1. Hardware resources will be consumed more but remain well below hardware capacity. Finally, it should be noted that the quality of the filtering with Solution 2 and Solution 3 are the same obtained during the software implementation of Section 3.

## 5. Conclusions

In this work, a new Modified Laplacian Vector Median Filter (MLVMF) for real-time denoising complex images corrupted by “salt and pepper” impulsive noise is proposed. The aim is to provide a real-time architecture capable of restoring the chest x-ray images as well as possible. This will allow experts to better diagnose possible pathologies such as cardiovascular diseases, asthma, lung cancer, pneumonia, COVID-19, and tumors. We propose two sets of modified Laplacian filters to identify corrupted pixels. The first set investigates images using a smaller rotation step compared to existing methods. This allows better detection of corrupted pixels. The second set of proposed filters allows the detection of adjacent noisy pixels. These pixels go unnoticed in front of conventional filters, which leads to a bad reconstruction of the image when the percentage of impulsive noise is high. New High-Level Synthesis (HLS) Designs for the proposed MLVMF Filter are presented. The proposed MLVMF shows better performances compared to VMF, VMRHF, and MSMF filters in terms of PSNR and NCD measurements. In fact, The MLVMF provides a high PSNR value of 5.5% and a low NCD value of 18.2%. A proposed Hardware implementation based on High-Level Synthesis (HLS) designs of the MLVMF enabled real-time processing allowing an execution time equal to 9 ms per 256 × 256 color image.

Our method allows, as shown by the experimental results, a very good detection of impulsive noise. However, the work presented here does not deal with the operation of replacing the corrupted pixel. To eliminate a noisy pixel after having identified it, we use the VMF approach. We believe that a more intelligent estimation of the true color of the noisy pixel is possible. However, this estimate can be costly in terms of resources and computation time. Future work will focus on this point because a better estimate of the true color of a corrupted pixel, added to the very good identification presented in this work, can bring us closer to the ideal denoising.

## Figures and Tables

**Table 1:** PSNR and NCD values of the VMF, VMRHF, MSMF, and MLVMF filters for various levels of impulsive noise.

Filter		VMF		VMRHF		MSMF		MLVMF	
Image	Noise	PSNR (dB)	NCD	PSNR (dB)	NCD	PSNR (dB)	NCD	PSNR (dB)	NCD
**Lena**	3%	32.340147	0.017115	32.782660	0.015688	35.472474	0.004766	37.423460	0.0042894
	5%	31.728372	0.018811	32.100663	0.017784	33.911660	0.008693	35.844625	0.0079106
	10%	29.577089	0.023299	29.734905	0.024349	30.400635	0.017641	32.087870	0.0159651
	20%	25.232852	0.041481	25.244443	0.052842	25.084609	0.049493	26.414093	0.0345932
	30%	20.856752	0.096637	21.130595	0.118374	20.657001	0.116282	21.731165	0.0955841
**Peppers**	3%	34.518151	0.012914	35.044101	0.011567	36.409296	0.005163	38.375398	0.0045951
	5%	33.217095	0.014426	33.596334	0.013441	34.123878	0.009285	36.068939	0.0084494
	10%	30.370972	0.018116	30.448538	0.019125	30.270752	0.020890	32.026456	0.0181743
	20%	25.730744	0.035530	25.695894	0.046225	25.121437	0.057862	26.477995	0.0314972
	30%	21.196992	0.083353	21.466820	0.103619	20.905560	0.117288	22.034460	0.0764975
**CXR1**	3%	33.637944	0.007440	33.797730	0.008316	35.523118	0.002363	37.476889	0.0021267
	5%	32.265345	0.008059	32.343503	0.010048	33.248306	0.004593	35.209956	0.0041796
	10%	29.716387	0.011066	29.690418	0.016741	29.858881	0.011025	31.590696	0.0099776
	20%	24.991928	0.027388	24.956495	0.046715	24.704409	0.038274	25.939629	0.0274849
	30%	20.404829	0.081102	20.680126	0.113743	20.243625	0.101027	21.296294	0.0717325
**CXR2**	3%	35.571872	0.009196	35.826513	0.007403	36.795335	0.003027	38.782283	0.0026940
	5%	33.170338	0.010195	33.264613	0.008741	33.436260	0.005518	35.342127	0.0050214
	10%	30.882192	0.011984	30.774950	0.013983	30.471932	0.011975	32.239304	0.0104183
	20%	25.765780	0.025517	25.680567	0.040658	25.238697	0.033607	26.601587	0.0239102
	30%	20.448200	0.079880	20.697093	0.112506	20.281939	0.091909	21.377164	0.0734534
**CXR3**	3%	37.002201	0.004489	37.195887	0.004118	38.126856	0.002395	40.223833	0.0021555
	5%	34.776916	0.005016	34.799607	0.005191	35.126686	0.004735	37.128907	0.0043089
	10%	31.703067	0.006751	31.494476	0.009411	31.463504	0.010426	33.209728	0.0054355
	20%	26.207766	0.021795	26.062978	0.038572	25.760547	0.036464	27.125856	0.0188541
	30%	21.351850	0.077362	21.600711	0.107416	21.129428	0.097884	22.228158	0.0738787
**Average**	**-**	**28.906630**	**0.029957**	**29.044420**	**0.038663**	**29.350670**	**0.034503**	**30.970270**	**0.0253275**

**Table 2:** FPGA resources and performances of the MLVMF-designed architectures.

	LUT	FF	BRAM_18K	DSP48E	Cycles
**Solution1**	10,687 (4%)	6590 (1%)	125 (7%)	13 (0.5%)	209,546,135
**Solution2**	43,343 (16%)	42,228 (8%)	125 (7%)	650 (26%)	5,304,902
**Solution3**	45,939 (17%)	43,685 (8%)	135 (8%)	665 (26%)	1,135,576
